# Elastic Stable Intramedullary Nailing Versus Plate Internal Fixation for Pediatric Diaphyseal Femur Fractures: A Systematic Review and Meta-analysis

**DOI:** 10.1007/s43465-024-01125-3

**Published:** 2024-03-16

**Authors:** Wanlin Liu, Wenqiang Li, Rui Bai, Xiangyu Xu, Zhenqun Zhao, Yan Wang

**Affiliations:** 1 Department of Orthopaedic, The Second Affiliated Hospital of Inner Mongolia, Medical University, Hohhot, 010030 Inner Mongolia China; 2 Department of Inner Mongolia Medical University, Hohot, 010030 Inner Mongolia China

**Keywords:** Elastic stable intramedullary nailing, Plate, Pediatric, Diaphyseal femur fractures

## Abstract

**Background:**

Elastic stable intramedullary nailing (ESIN) and plates are currently the main internal fixation for treating Pediatric Diaphyseal Femur Fractures (PDFF), and the optimal choice of internal fixation is controversial. The purpose of this meta-analysis is to compare the surgical outcomes and complications of the two fixation methods.

**Materials and Methods:**

MEDLINE, Embase, and the Cochrane Library were systematically searched for studies published up to March, 2023, that compared ESIN and plate fixation techniques for treating PDFF. Pooled analysis identified differences in surgical outcomes between ESIN and plate, mainly regarding surgical outcomes and postoperative complications, such as time at surgery, fracture healing time, blood loss and related complications.

**Results:**

We included 10 studies with 775 patients with PDFF in our review. Of these, 428 and 347 were treated with ESIN and Plate, respectively. In terms of postoperative complications, ESIN led to a shorter surgery time [MD = − 28.93, 95% CI (− 52.88 to − 4.98), *P* < 0.05], less blood loss [MD = − 66.94, 95% CI (− 87.79 to − 46.10), *P* < 0.001] and more fracture healing time [MD = 2.65, 95% CI (1.22–4.07), *P* < 0.001]. In terms of postoperative complications, ESIN led to fewer fections (RR = 0.77, 95% CI 0.37, 1.60, *P* = 0.48), fewer angulation deformities (RR = 0.80, 95% CI 0.35, 1.83, *P* = 0.60) and more prominent implants (RR = 3.36, 95% CI 1.88, 6.01, *P* < 0.001), more delayed unions (RR = 4.06, 95% CI 0.71, 23.06, *P* = 0.11).

**Conclusions:**

ESIN and Plate have similar rates of complications besides a prominent implant rate, while ESIN has a shorter period of operation and less intraoperative bleeding. Although both options are suitable, the results of this study support the use of ESIN rather than plates in the treatment of PDFF in terms of complication rates. In clinical applications, surgeons should choose the appropriate treatment method according to the actual situation.

## Introduction

Pediatric diaphyseal femur fractures (PDFF) are common in children's orthopedics at different ages, accounting for 1.4–1.7% of child fractures [1, 2]. When choosing the appropriate treatment, several factors must be taken into account the patient’s weight, fracture type, and type of injury. There are various fixation techniques for femoral shaft fractures, including direct Spica casting and Spica casting after traction, rigid intramedullary nailing and elastic stabilized intramedullary nailing (ESIN), open plates, and submuscular plates. However, the preferred method of operative fixation for these fractures is not clear in clinical practice guidelines.

According to the clinical practice guidelines of the American Academy of Orthopedic Surgeons (AAOS), elastic stable intramedullary nailing (ESIN) is recommended for children aged 5–11. Although widely accepted, ESIN has been linked to suboptimal outcomes in pediatric femoral fractures characterized by biomechanical instability and significant hardware-related adverse events. The higher incidence of complications by ESIN (from 10 to 80%), including infection, prominent implant, unplanned reoperations, differences in limb length, and deformity healing, was aggravated in children. In general, treatment is age-dependent and there is a substantial overlap between age groups. According to studies, elastic intramedullary nails are appropriate for patients weighing less than 50 kg and children aged 6–15 [3, 4]. Due to its good bone reconstruction potential, ESIN has been a common treatment for stable femoral fractures in children for a long time [5].

There is growing evidence that these fractures can also be treated with open or submuscular plates which have emerged as a new method of treating femoral shaft fractures in children. Many studies have concluded that there is no difference in intraoperative and postoperative complications between open plates and submuscular plates [6, 7]. The treatment of pediatric femoral diaphyseal fractures by incised reduction plate fixation reduces the complication rate and results in a faster healing rate and early weight bearing. In comminuted and unstable fractures of the femoral stem, plates provide good results compared to flexible intramedullary nailing. Although the weight of the children was not specified in the literature included in this study, the age of the included children was 4–15 years old.

The exact therapeutic effectiveness of elastic stable intramedullary nailing and plates is still controversial. Over the past two decades, there have been some randomized controlled studies and retrospective studies on ESIN and plate fixation of femoral fractures. However, the sample quantity and methodological quality of these studies are limited. Thus, we conducted a meta-analysis to evaluate the outcome of ESIN with plate fixation treatment in children aged 4–15 years. Analysis of outcomes included time at surgery, fracture healing time, blood loss and related complications.

## Materials and Methods

Bias risk assessment at the research level, using the Cochrane Bias Risk Tool (RoB) to assess bias risk in randomized controlled trials (RCT) and using the Methodological Index for Nonrandomized Studies (MINORS) to assess bias risk in retrospective study (RCS). The Cochrane bias risk assessment tool includes subsequent areas: Sequence Generation (Selection Deviation), Assignment Selection Hidden (Selection deviation), blind vision of participants and personnel (Performance Bias), Blindness of Result Evaluation (Detection deviation), insufficient result data (loss deviation), selective results reports (reporting deviations) and other possible causes of deviation. The authors' interpretations are classified as “low risk,” “high risk” or “biased risk of ambiguity.”

Outcome indicators included:

1. Angulation deformity was defined as greater than 10° angulation in the sagittal plane (flexion or extension) or greater than 5° angulation in the coronal plane (varus or valgus).
2. Delayed union was defined as persistent bone pain and tenderness 3 months after the fracture with no complete healing on imaging. Nonunion was defined as Nonunion was defined as the absence of osseous union more than 6 months after the injury.

## Results

### Study Selection

A systematic search of PubMed, Embase, Web of Science, Cochrane Library, and Google database identified 620 potentially eligible studies, and no additional records were found during manual searches of reference lists.

**Fig. 1:**
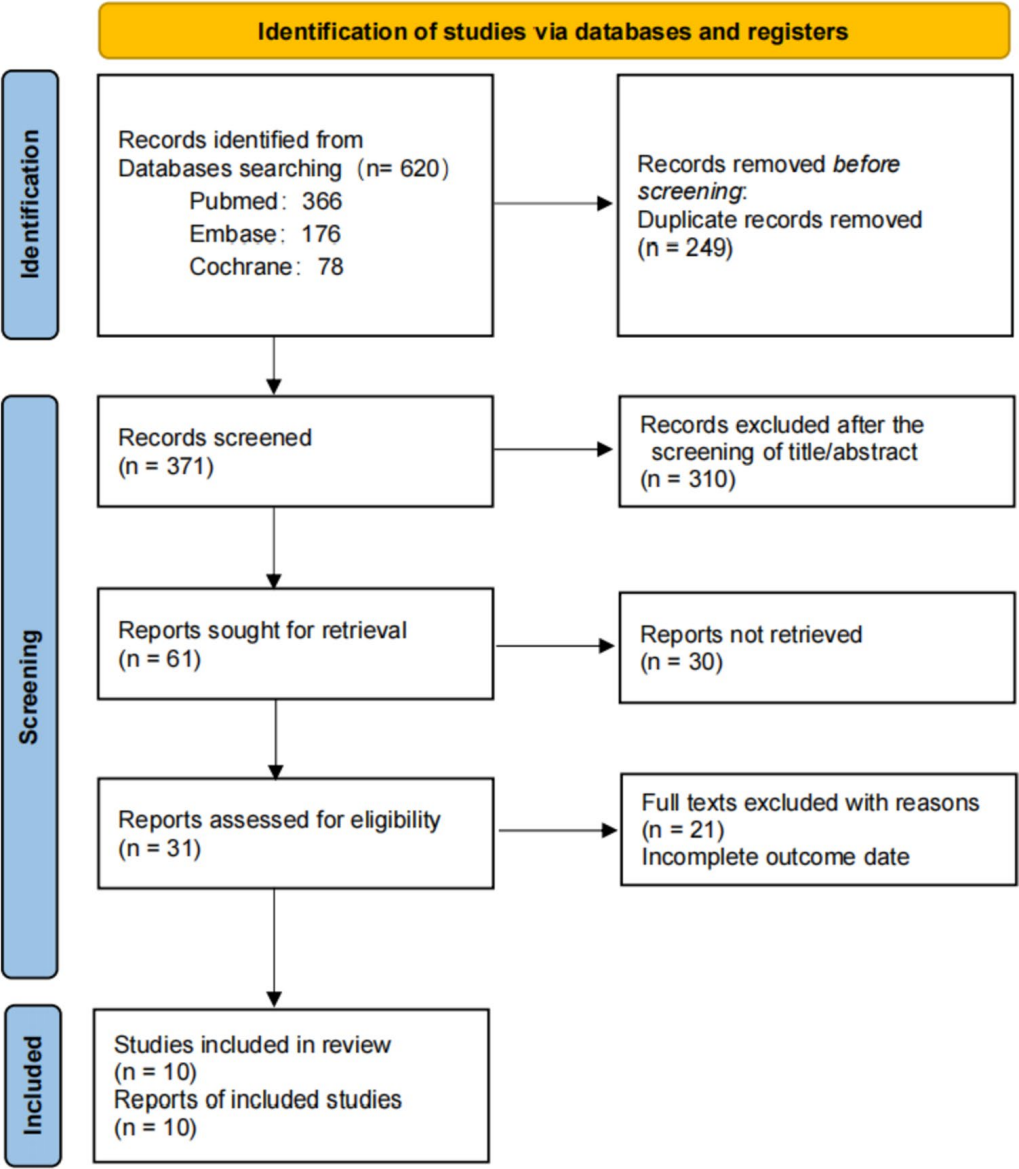
PRISMA flow diagram

After removing duplicates and ineligible records following an automated tool, we screened the remaining 371 studies. Of these, 340 articles were left for full-text review, and 21 articles were excluded according to the inclusion and exclusion criteria. Finally, 4 RCT studies [8–11], 5 retrospective studies [12–16], and 1 prospective study [17] were included in the review. The literature screening process (PRISMA diagram) is shown in Fig. 1.

### Patient Characteristics

The meta-analysis included 4 RCT studies, 5 retrospective studies, and 1 prospective study. Table 1 summarizes the general information included in the publications. A total of 775 cases of femoral fractures in children were retrieved from 10 studies published from 2018 to 2023. Of these, 428 cases (55.2%) were treated with flexible intramedullary nails (ESIN) and 347 cases (44.8%) were treated with Plates.

**Table 1:** Clinical characteristics of included studies

Study	Study period	Design	Region	Age range (years)	Sex (male/female)	Cases (ESIN/plate)	Follow-up	Fracture type	MINORS score
Wang et al. [8]	2016–2018	RCT	China	4–15	74/46	60/60	Not stated	Sein-sheimer classification:40 cases ofType-II, 10 cases of Type-III and 10cases of Type-IV	
Wael [10]	2018–2020	RCT	Egypt	6–12	40/10	25/25	12 months	AO/OTA (ESIN/SMP): A3 simpletransverse < 30°: 18 (72%)/16 (64%) A2simple oblique > 30°: 7 (28)/9 (36%)	
James et al. [9]	2013–2016	RCT	India	6–15	26/14	20/20	24 months	AO/OTA (ESIN/SMP): Transverse: 13/15 Short oblique: 7/6	
Hayat [11]	2014–2016	RCT	Pakistan	6–12	78/24	51/51	At least 12 months	Not stated	
Venkataraman et al. [17]	2018–2020	PCS	India	6–14	39/20	31/28	36 weeks	Transverse: 22 (37.3%), comminuted: 13 (22%), oblique: 18 (31%), spiral: 6 (10%)	20
Allen et al. [15]	2004–2014	RCS	America	5–11	45/20	50/15	Not stated	Not stated	18
Chen et al. [14]	2005–2017	RCS	America	7.7 ± 2	43/15	28/30	Mean follow-up 22 months	21 comminuted, 16 transverse, 13oblique and 8 spiral fractures	18
Li et al. [12]	2008–2018	RCS	China	5–11	71/51	77/45	At least 24 months	AO: 32-D/5.1 and 32-D/5.2	17
Bajelidze et al. [16]	2013–2017	RCS	Georgia	6–15	95/42	76/61	Mean follow-up 10.68 months	AO: A1-44 cases, A2-28 cases, A3-38cases, B1-6 cases; B2-2 cases; B3-2cases; C1-3 cases and C3-2 cases	15
Luo et al. [13]	2015–2017	RCS	China	5.9 ± 2.8	Not stated	10/12	At least 24 months	All comminuted fractures	18

### Quality Evaluation

**Fig. 2:**
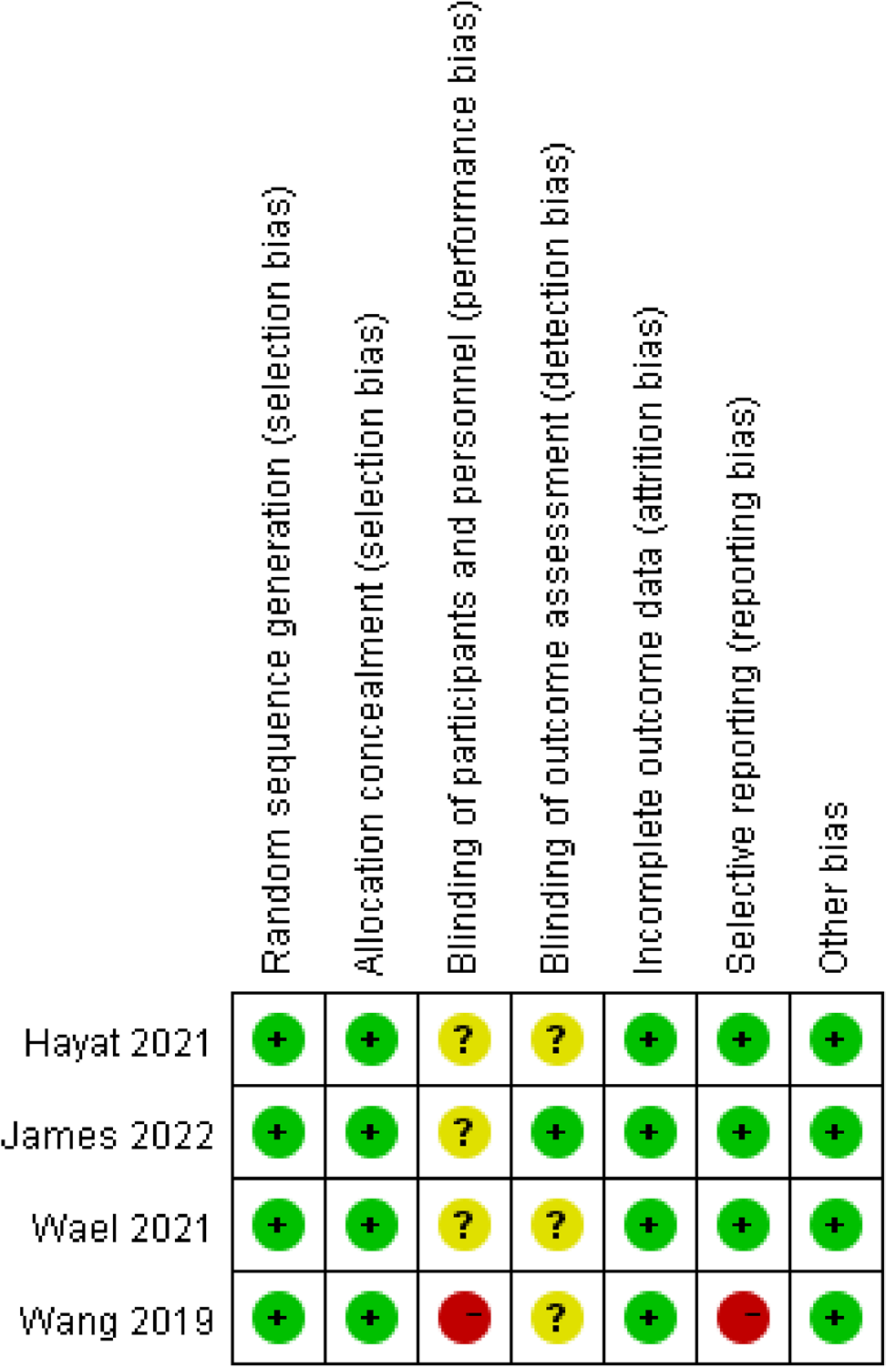
Risk of bias assessment (1)

**Fig. 3:**
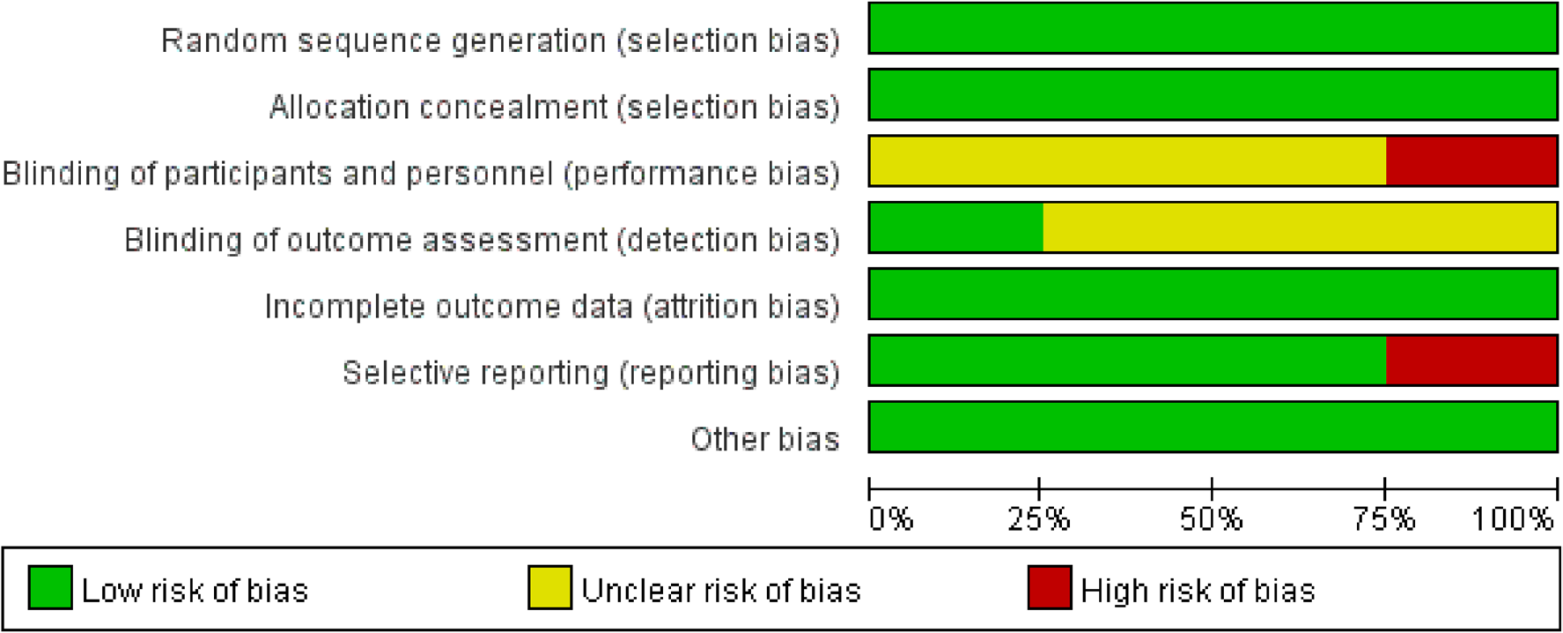
Risk of bias assessment (2)

According to the Cochrane Bias Risk Assessment Tool, the quality of the included studies ranged from medium to high quality. Therefore, the inclusion of research has acceptable quality for inclusion. Details of bias risks are summarized in Figs. 2 and 3.

### Outcome Indicators

#### Time at Surgery

**Fig. 4:**
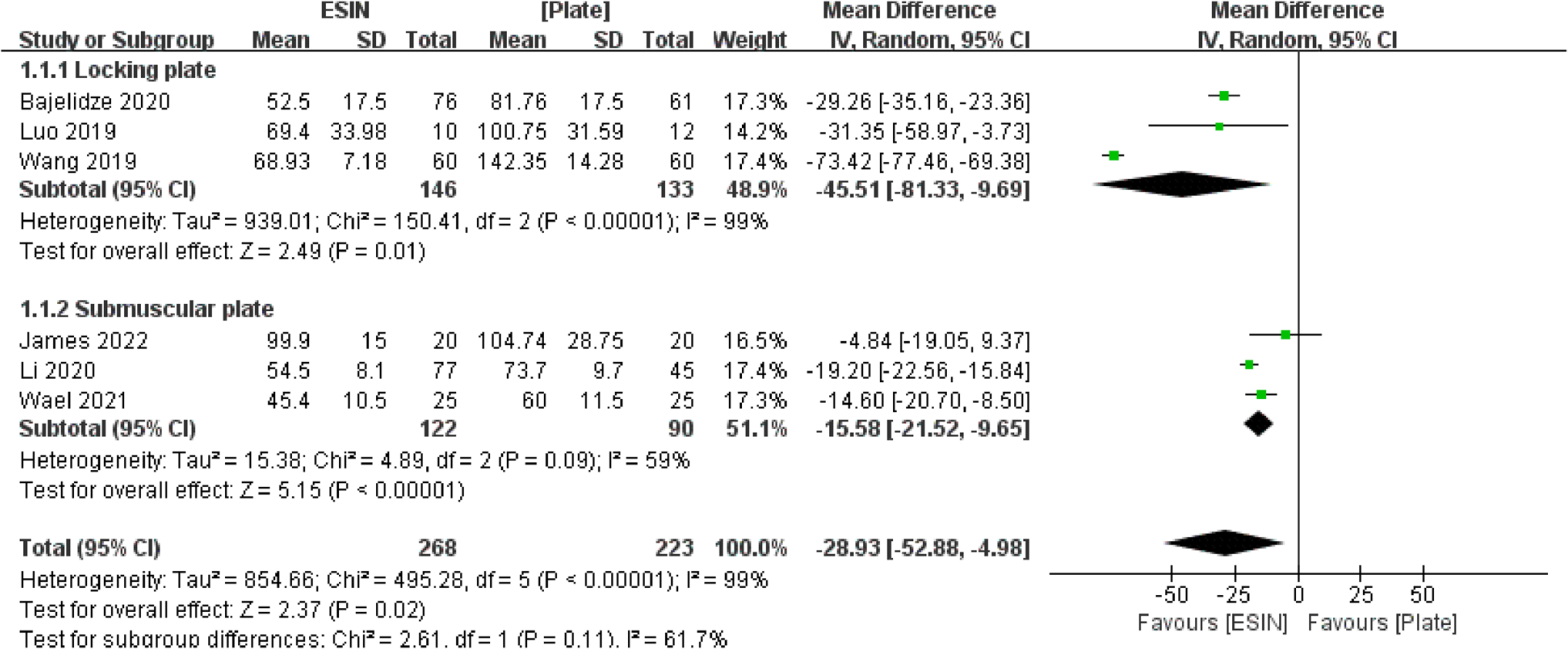
Forest plot: mean time at surgery in the ESIN group versus Plate group

Six studies compared the surgical time of treating pediatric diaphyseal femur fractures. In the subgroup of locking plate, there was a significant difference between the two fixation methods (MD = − 45.51, 95% CI [− 81.33 to − 9.69], *P* < 0.05). In the subgroup of submuscular plate, there was a significant difference between the two fixation methods (MD = − 15.58, 95% CI [− 21.52 to − 9.65], *P* < 0.001). The combined mean difference across all studies (MD = − 28.93, 95% CI [− 52.88 to − 4.98], *P* < 0.05). A large heterogeneity was observed between these studies (I^2^ = 99%, *P* < 0.001) (Fig. 4).

#### Blood Loss

**Fig. 5:**
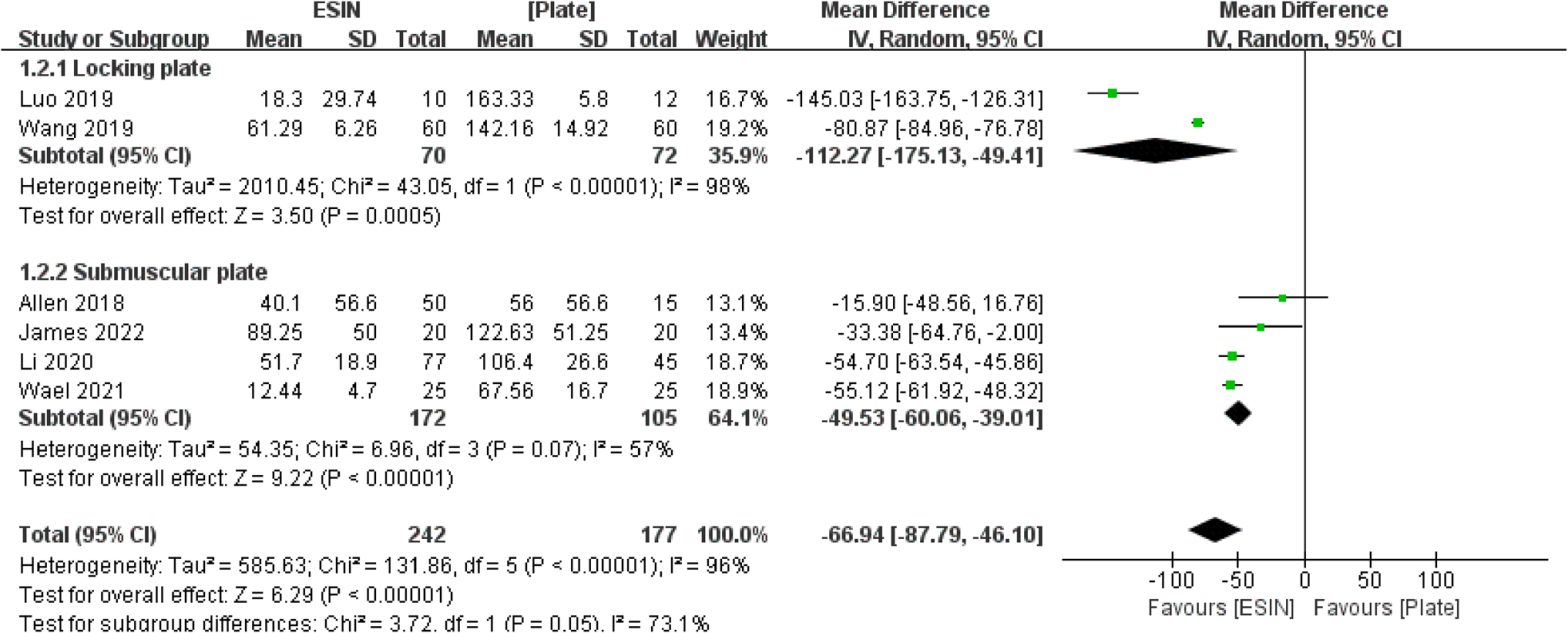
Forest plot: mean blood loss in the ESIN group versus Plate group

Six studies assessed blood loss in a total of 419 patients. The subgroup of locking plate reported a statistically significant difference favoring ESIN over the Plate group [MD = − 112.27, 95% CI (− 175.13 to − 49.41)]. The subgroup of submuscular plate similarly supported the ESIN group. There was still a large heterogeneity observed between these studies when combined, but the blood loss during the surgery was still in favor of ESIN with statistical significance (MD = − 66.94, 95% CI, [− 87.79 to − 46.10], I^2^ = 96%, *P* < 0.001) (Fig. 5).

#### Fracture Healing Time

**Fig. 6:**
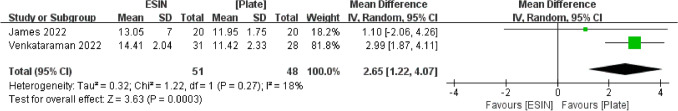
Forest plot: mean fracture healing time in the ESIN group versus Plate group

Two studies assessed the fracture healing time in a total of 99 patients. There was a statistically significant difference favoring Plate over the ESIN group (MD = 2.65, 95% CI [1.22 to 4.07], I^2^ = 18%, *P* < 0.001). The two fixation methods showed a significant difference (Fig. 6).

#### Weight

**Fig. 7:**
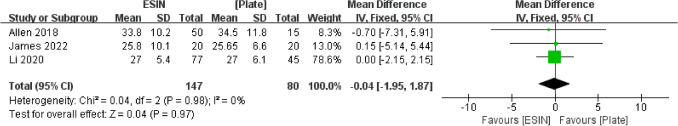
Forest plot: mean weight in the ESIN group versus Plate group

Three studies evaluated the weight of 227 patients at the time of injury. There were no significant differences in body weight among the patients included in the study, and there was no significant heterogeneity in the assessments (Fig. 7).

#### Infection

**Fig. 8:**
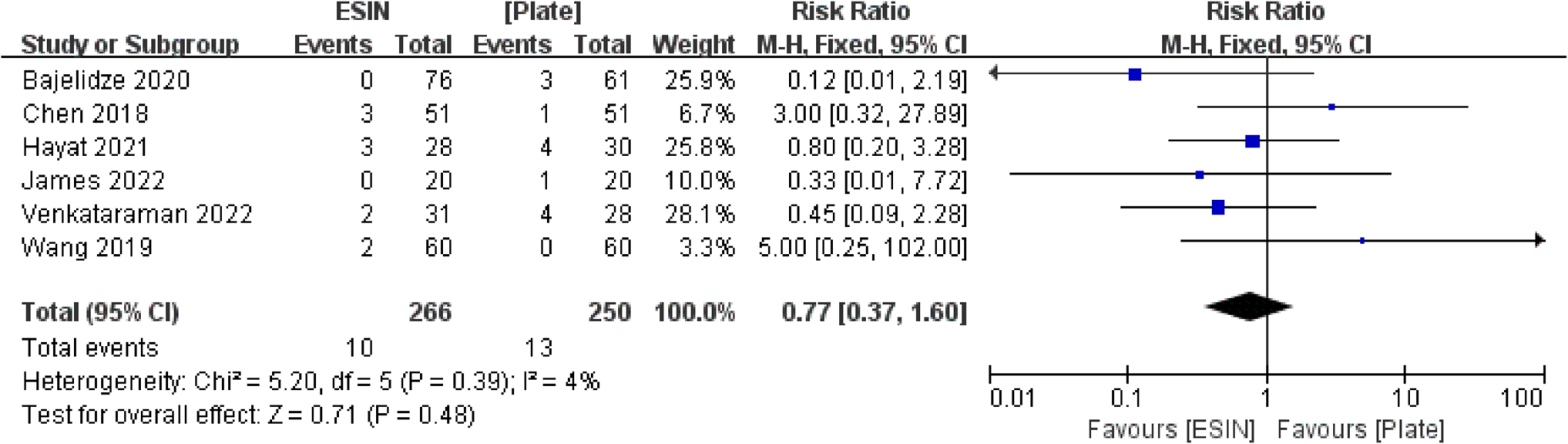
Forest plot: risk ratio of infection in the ESIN group versus Plate group

All included trials of 516 children reported the infection in the analysis, 266 in the ESIN group and 250 in the Plate group. Ten children (3.8%) in the ESIN group and thirteen children (5.2%) in the Plate group had infections. There are deep infections and superficial infections, but none of these require surgical management. There was no statistically significant difference between the two procedures, with low heterogeneity (RR = 0.77, 95% CI 0.37, 1.60, I^2^ = 4%, *P* = 0.48) (Fig. 8).

#### Prominent Implant

**Fig. 9:**
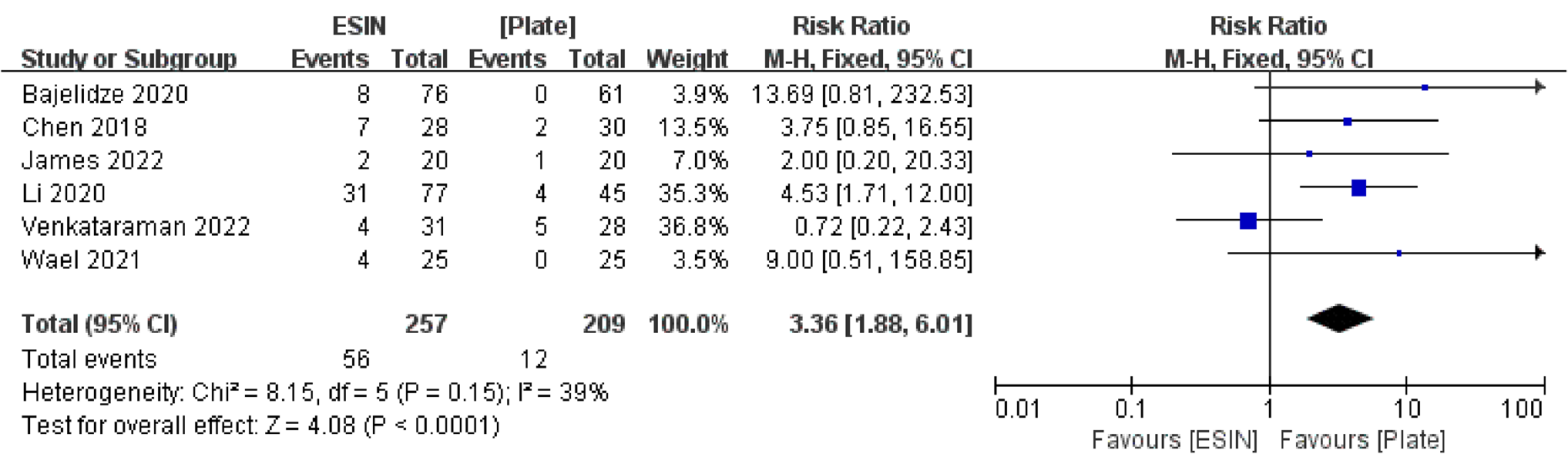
Forest plot: risk ratio of prominent implant in the ESIN group versus Plate group

Six studies of 466 children were included in the prominent implant analysis in which fifty-six (21.8%) of 257 patients were treated with elastic stable intramedullary nailing and twelve (5.7%) of 209 patients were treated with plates with prominent implants. Analysis of the incidence of the prominent implant shows that the ESIN group is higher, and the statistical analysis showed a statistically significant difference between the two fixation methods with low heterogeneity (RR = 3.36, 95% CI 1.88, 6.01, I^2^ = 39%, *P* < 0.001) (Fig. 9).

#### Angulation Deformity

**Fig. 10:**
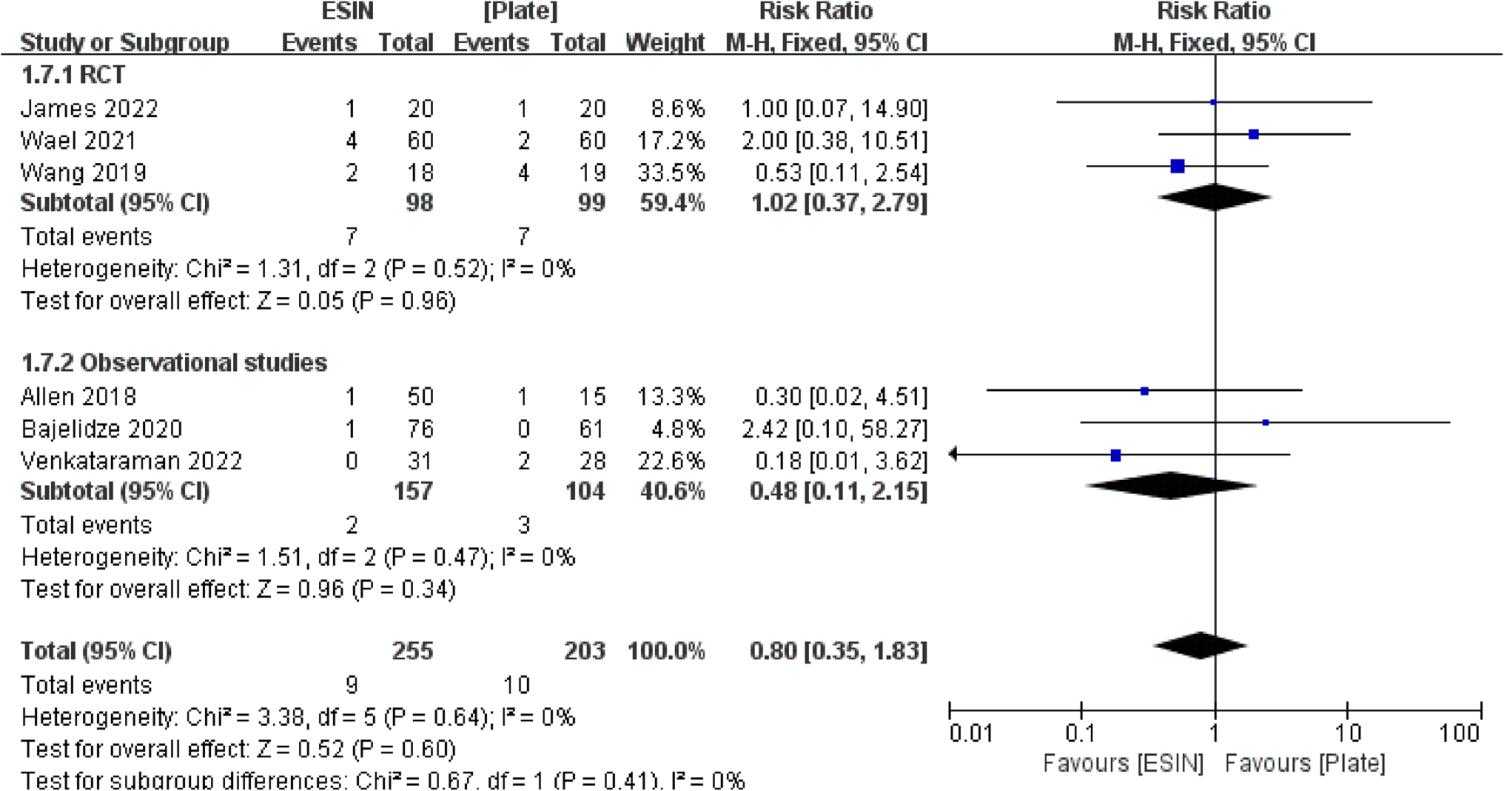
Forest plot: risk ratio of angulation deformity in the ESIN group versus Plate group

Six studies involving 458 children were included in the angulation deformity analysis. Angulation deformity occurred in nine (3.5%) of 255 children treated with ESIN and in ten (4.9%) of 203 patients treated with Plate. Patients subjected to Plate showed a higher occurrence of angulation deformity compared to those who received ESIN treatment, but the statistical analysis showed no difference between these patients (RR = 0.80, 95% CI 0.35, 1.83, *P* = 0.60) (Fig. 10).

#### Delayed Union and Nonunion

**Fig. 11:**
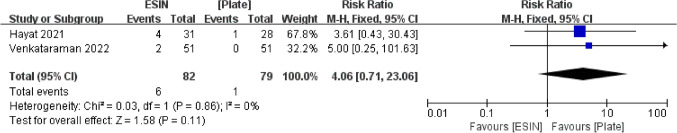
Forest plot: risk ratio of delayed union and nonunion in the ESIN group versus Plate group

Three studies of 198 children were included in the delayed union rate analysis. Delayed union and nonunion occurred in six (7.3%) of 82 children treated with ESIN and in one (1.3%) of 79 patients treated with Plate. The statistical analysis showed no difference between the two fixation methods with low heterogeneity (RR = 4.06, 95% CI 0.71, 23.06, I^2^ = 0%, *P* = 0.11) (Fig. 11).

## Discussion

The main result of this pooled analysis was that there was no apparent difference between the ESIN and plate groups in the incidence of postoperative primary complications of infection (3.8% vs. 5.2%; *P* = 0.48), angulation deformity (3.5% vs. 4.9%; *P* = 0.60), and delayed union and nonunion (7.3% vs. 1.3%; *P* = 0.11). Our study showed that the prominent implant rate (21.8% vs. 5.7%; *P* < 0.001) was significantly higher in patients treated with ESIN than in those treated with plates. The higher incidence of prominent implants in ESIN is similar to previous studies that have shown implant irritation rates of up to 52% [18]. Major and minor complication rates for plating pediatric femoral fractures are 6% and 7% respectively [19]. However, the incidence of complications by ESIN was 62% [20]. A review found a significantly increased relative risk of all complications, particularly malunion and prominent implants, when fractures in this cohort were treated with ESIN compared with plates [21]. This review partially differs from our study which found no significant difference in the incidence of deformity in PDFF treated with these two fixation modalities. Allen et al. [15] are more supportive of the use of ESIN because the use of plates resulted in higher estimated blood loss, longer operative times, higher costs and equivalent pain compared to ESIN. However, in the study of Sutphen et al. [22], plates are associated with minimal complication rates, the fastest times to union, and full weight bearing when compared to flexible intramedullary nailing, even though there is limited high-quality evidence.

The fracture type may also have an impact on the incidence of postoperative deformities, but this study was not grouped according to fracture stability. In the past, unstable pediatric femoral diaphyseal fractures have been treated with plate fixation with good results [23, 24]. The study by Sink et al. [20] concluded that elastic stable intramedullary nailing has a high and serious complication rate in the treatment of PDFF, and treatment options other than flexible intramedullary nails should be considered. However, several recent studies [25, 26] have found that there was no significant difference in the complication rate between length stable and unstable fractures treated with ESINs. ESINs and plate techniques are equally suitable for unstable fracture types with no effect on outcome. Further clinical evidence, such as large multiregional clinical trials, is needed to support our finding.

In this meta-analysis, the plate was associated with a longer operative time and more blood loss than ESIN. Plate treatment in femur shaft fracture surgery increases the exposed surface area and blood loss, thus increasing the risk of infection. However, our meta-analysis showed that the infection rate was not statistically significant between the two fixation groups. In addition, the fracture healing time was significantly shorter in the Plate group than in ESIN group. A biomechanical study [27] suggests that using locked plating for pediatric femur fracture models results in a more stable biomechanical structure compared to elastic intramedullary nailing. There is also a biomechanical study [28] that revealed that patients weighing over 40–45 kg are vulnerable to losing sagittal and coronal plane resurfacing when titanium elastic nails are used to stabilize transverse fractures of the mid-femur. According to studies, femoral shaft fractures in children and adolescents weighing 50 kg or more and over the age of ten have an increased rate of complications [29]. Weight is not independently associated with adverse outcomes when age is also included in logistic regression [30]. Although the literature we included does not limit the types of fracture and the weight range of patients included in the literature is 30–60 kg, these two fixed methods have no statistical significance in weight or the incidence of angulation deformity.

According to the authors, this is the first systematic review of the differences between ESIN and plate internal fixation in the treatment of femoral shaft fractures in children. Most of our results were consistent with previous meta-analyses, except for the results of a comparison of time at surgery, blood loss, and weight. In the meta-analysis of ESIN and Plate, several outcomes were more heterogeneous. As a result, the total average difference may not seem reliable. However, none of these results contradict each other. Nevertheless, these results should be interpreted with caution.

We encountered some limitations in analyzing the results. First, there are very few randomized controlled trials. Second, due to the limited number of studies included, it is not possible to conduct a publication bias analysis, which may be biased. Third, due to the characteristics of the meta-analysis, we could not control for fracture severity as a confounding factor. confounding factor, despite our best efforts to reduce the bias of each included study. Additionally, the varied weights and fracture types that can have an impact on the outcomes were not reported in this meta-analysis. Fortunately, most of the results of this meta-analysis show that the heterogeneity between the included studies is low, indicating that the meta-analysis conclusions are stable and relatively reliable.

In conclusion, based on current evidence, ESIN and Plate have similar rates of complications in addition to a prominent implant rate, while ESIN has a shorter period of operation and less intraoperative bleeding. Although both options are suitable, the results of this study support the use of ESIN rather than plate in the treatment of PDFF in terms of complication rates. In clinical applications, surgeons should select the appropriate treatment method according to the actual situation. Further study should concentrate on how these two fixations affect the clinical prognosis of children with various body weights and fracture types, as well as on the time spent on weight bearing in patients with these two fixations.

## Data Availability

The datasets generated during and/or analyzed during the current study are available from the corresponding author on reasonable request.
